# The Influence of Food Intake and Preload Augmentation on Cardiac Functional Parameters: A Study Using Both Cardiac Magnetic Resonance and Echocardiography

**DOI:** 10.3390/jcm12216781

**Published:** 2023-10-26

**Authors:** Lasse Visby, Rasmus Møgelvang, Frederik Fasth Grund, Katrine Aagaard Myhr, Christian Hassager, Niels Vejlstrup, Raj Mattu, Charlotte Burup Kristensen

**Affiliations:** 1 Department of Cardiology, The Heart Center Rigshospitalet, Blegdamsvej 9, DK-2100 Copenhagen, Denmarkfrederikgrund@gmail.com (F.F.G.);; 2 Institute of Clinical Medicine, Faculty of Health and Medical Sciences, University of Copenhagen, Blegdamsvej 3B, DK-2100 Copenhagen, Denmark; 3 Cardiovascular Research Unit, University of Southern Denmark, Baagoees Allé 15, DK-5700 Svendborg, Denmark; 4 Kettering General Hospital NHS Foundation Trust, University Hospitals Northamptonshire, Kettering NN1 5BD, Northamptonshire, UK; 5 University College London, Gower St., London WC1E 6BT, UK; 6 Department of Cardiology, Lund University Hospital, Entrégatan 7, SE-22242 Lund, Sweden

**Keywords:** heart failure, transthoracic echocardiography, cardiac magnetic resonance, hemodynamic, food intake, preload augmentation

## Abstract

(1) Background: To investigate how food intake and preload augmentation affect the cardiac output (CO) and volumes of the left ventricle (LV) and right ventricle (RV) assessed using cardiac magnetic resonance (CMR) and trans-thoracic echocardiography (TTE). (2) Methods: Eighty-two subjects with (*n* = 40) and without (*n* = 42) cardiac disease were assessed using both CMR and TTE immediately before and after a fast infusion of 2 L isotonic saline. Half of the population had a meal during saline infusion (food/fluid), and the other half were kept fasting (fasting/fluid). We analyzed end-diastolic (EDV) and end-systolic (ESV) volumes and feature tracking (FT) using CMR, LV global longitudinal strain (GLS), and RV longitudinal strain (LS) using TTE. (3) Results: CO assessed using CMR increased significantly in both groups, and the increase was significantly higher in the food/fluid group: LV-CO (ΔLV-CO: +2.6 ± 1.3 vs. +0.7 ± 1.0 *p* < 0.001), followed by increased heart rate (HR) (ΔHR: +12 ± 8 vs. +1 ± 6 *p* < 0.001). LV and RV achieved increased stroke volume (SV) through different mechanisms. For the LV, through increased contractility, increased LV-EDV, decreased LV-ESV, increased LV-FT, and GLS were observed. For the RV, increased volumes, increased RV-EDV, increased RV-ESV, and at least for the fasting/fluid group, unchanged RV-FT and RV-LS were reported. (4) Conclusions: Preload augmentation and food intake have a significant impact on hemodynamic and cardiac functional parameters. This advocates for standardized recommendations regarding oral intake of fluid and food before cardiac assessment, for example, TTE, CMR, and right heart catheterization. We also demonstrate different approaches for the LV and RV to increase SV: for the LV by increased contractility, and for the RV by volume expansion.

## 1. Introduction

Assessment of hemodynamic and systolic function of the heart is fundamental for diagnosis, follow-up, and prognosis in patients with or at risk of cardiac dysfunction [1,2,3,4]. Transthoracic echocardiography (TTE) is the most commonly used method; however, cardiovascular magnetic resonance (CMR) is acknowledged as the gold standard for the measurements of volumes and functions of both the left and right ventricles [5].

Previous studies have examined the effect of food intake on left ventricular (LV) hemodynamic parameters, such as cardiac output (CO), stroke volume (SV), and heart rate (HR), using conventional TTE and Tissue Doppler Echocardiography (TDE). These demonstrated that food intake significantly alters the hemodynamics and function of the LV [6,7,8,9,10,11]. CMR and TTE are readily available and provide feasible, reproducible assessments of cardiac function using feature tracking (FT) and global longitudinal strain (GLS), respectively [12,13]. Since the anatomy and function of the right ventricle (RV) differ from the LV, it is inappropriate to extrapolate our knowledge of the LV to the RV [14]. Furthermore, there is a paucity of investigation of the combined effects of preload augmentation and food intake on both the RV and LV using CMR to assess function and hemodynamics.

Accurate assessment of cardiac function and hemodynamics is important in patients scheduled for sequential examinations, such as monitoring medical therapy in heart failure, and assessments prior to or during cancer treatment, such as chemotherapy or immunotherapy [5,15,16,17]. Furthermore, TTE can play an important role in cardiovascular and hemodynamic assessment in a perioperative and intensive care setting, where it has the potential to reveal cardiovascular issues and guide fluid therapy [18,19,20,21].

While patients scheduled for stress testing by CMR are instructed to abstain from caffeine intake, patients scheduled for echocardiography, CMR without stress testing, and right heart catheterization do not routinely receive recommendations regarding oral intake prior to the assessment of cardiac function. Food intake and preload vary throughout the day and between days, so they may potentially affect the results and precipitate inappropriate changes in care.

The purpose of this study is to investigate how food intake and preload augmentation affect the function and volumes of the LV and RV assessed using CMR and strain by TTE.

## 2. Materials and Methods

### 2.1. Study Design

**Figure 1:**
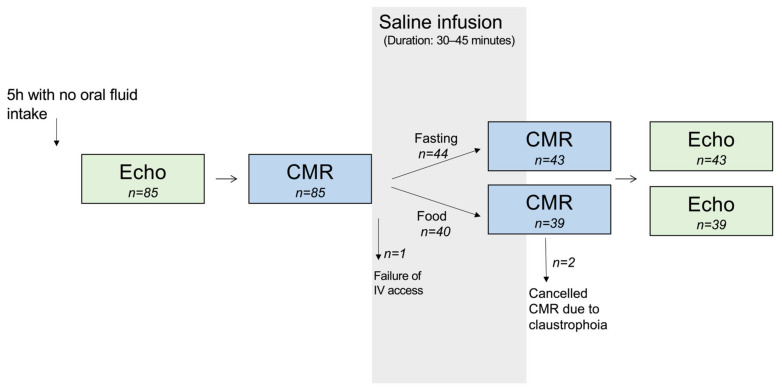
Study design. CMR: cardiovascular magnetic resonance; Echo: echocardiography; IV: intravenous.

All subjects scheduled for routine TTE at the Echocardiography Laboratory at the Department of Cardiology, Rigshospitalet, Denmark, were sequentially invited to participate in the study over a period of 15 months. The only inclusion criteria were age ≥18 years and sinus rhythm on the examination day. Exclusion criteria were heart failure with New York Heart Association (NYHA) class III–IV, pregnancy, breastfeeding, or any contraindication to CMR. The study design is an observational cross-sectional study and is summarized in Figure 1. Patients were asked for inclusion on days where the study coordinator was present, and 85 agreed to participate, including subjects with and without known cardiac disease. This incorporated hypertrophic cardiomyopathy, moderate/severe aortic valve stenosis or aortic valve regurgitation, dilated cardiomyopathy, acromegaly, Marfan, muscular dystrophia, and arrhythmogenic right ventricular cardiomyopathy. One subject was excluded on the examination day because of failure with intravenous (IV) access, and two subjects were excluded because the second CMR examination was interrupted because of claustrophobia. The final study population consisted of 82 subjects.

The patients were divided into two subgroups: (1) food/fluid and (2) fasting/fluid. The first 50% of the patients were categorized in the food/fluid group, and the last 50% in the fasting/fluid group. The food/fluid group had a meal representing a normal lunch immediately after the pre-examinations during the fluid infusion. The infusion and food period for the food/fluid group lasted 30–45 min. The fasting/fluid group was not allowed any oral intake and kept fasting before and during the entire examination.

All subjects were instructed to abstain from any oral intake and cigarette smoking five hours prior to the examination. In this way, the baseline for all the patients was as equal as possible, and we avoided the risk that cigarette smoking could affect the hemodynamics, hence the results [22,23]. The baseline examination consisted of TTE, followed immediately by CMR. Thereafter, isotonic saline was administered intravenously over approximately 45 min (median 2 L). A total of 19 subjects were administered reduced infusions due to an increased risk of pulmonary edema, and one subject was administered an increased infusion due to a large body surface area (BSA).

To investigate the impact of food intake on cardiac function and hemodynamics, the patients were divided into two subgroups: (1) food/fluid and (2) fasting/fluid. The food/fluid group had a meal representing a normal lunch consisting of a sandwich, soda, and coffee/tea during the fluid infusion. The fasting/fluid group was not allowed any oral intake and kept fasting before and during the entire examination.

The second CMR and echocardiography were performed immediately after the infusion of 1.5 L saline, while the remaining 0.5 L was infused at a slower rate during the CMR. All examinations were performed within 1.5 h after the saline infusion. HR was measured at 4 points: before the examination, during the main fluid infusion, right after the second CMR, and right after the last TTE. The saline infusion index was calculated as the ratio of saline infusion to the BSA. The investigation conforms with the principles outlined in the Declaration of Helsinki and was approved by the local ethics committee of the Capital Region of Denmark (Protocol number H-16029778, date 27 September 2016). All subjects provided written, informed consent.

### 2.2. CMR Acquisition and Analysis

CMR images were recorded using a 1.5 T Imaging System (GE Health Care Optime MR450W; GE Healthcare, Waukesha, WI, USA) and a phased-array cardiac coil. Cine images were acquired during breath-hold and steady-state free precession cine sequences with retrospective gating. Slice thickness was 8 mm, there were no gaps, the field of view was 300–360 mm, and there were 25 phases/cardiac cycles. Offline analysis was performed using CVI42 (Circle Cardiovascular Imaging Inc., Calgary, AB, Canada) and blinded for all other information, including fluid and food status. End-diastole and end-systole were identified as the phases with the largest and smallest volumes, respectively. For volumetric measurements of the LV, the endocardial contours were manually delineated in the short-axis stack and for the RV in the transversal stack. The LV papillary muscles were considered part of the LV volume. Feature tracking (FT) was performed in the long-axis views using two-chamber, three-chamber, and four-chamber views. The adequacy of FT was assessed by visual evaluation of strain curves and whether the contours followed the myocardium. Cardiac output was calculated as SVxHR. Indexed CO was calculated by dividing measured CO with BSA. LV geometry was assessed by dividing patients into four groups: normal, hypertrophic, dilated, and dilated + hypertrophic based on the CMR examination [24].

### 2.3. Echocardiographic Acquisition and Analysis

Two-dimensional (2D) TTE images were acquired by one experienced sonographer using a Vivid E95 ultrasound scanner (GE Healthcare Vingmed Ultrasound AS, Horten, Norway) and a M5Sc-D matrix phased array transducer (1.5–4.6 MHz). A dataset including TTE of both LV and RV for GLS analysis was obtained. TTE examinations were stored externally and analyzed offline using EchoPac software version 201 (GE Vingmed, Horten, Norway). LV GLS was evaluated from basal, mid, and apical segments of apical two-, three-, and four-chamber (2CH, 3CH, 4CH) views. RV strain was obtained in the 4CH view, assessing strain for both the free wall and for all six segments, including the septum. Adequate image quality for LV strain was defined as ≥4 trackable segments in each view, whereas adequate RV strain quality was defined as ≥5 trackable segments for the complete RV strain analysis and all three segments for the free wall analysis [25].

### 2.4. Statistics

Statistical analysis was performed using SPSS Statistics for Macintosh (Version 28.0; Armonk, NY, USA; IBM Corp.). Continuous variables are presented as mean ± SD, and categorical variables as frequencies and percentages. Comparisons between groups were performed using two-tailed paired and unpaired *t*-tests for normally distributed variables. To test the effects of food intake and fluid infusion on the LV and RV CO and FT on CMR and echocardiographic strain, multiple linear regression was performed. Friedman’s test was used to test changes in HR over time. To assess the risk of underestimating or overestimating GLS at subsequent examinations if the patient eats/drinks at first and not at the second examination, the post-examination was defined as baseline, and differences between post-GLS and pre-GLS were tested with a chi-square test and compared with current cardio-oncology guidelines. Statistical significance was defined as *p* < 0.05.

## 3. Results

### 3.1. Study Population

The baseline characteristics of the two study groups, the fasting/fluid group and the food/fluid group, are summarized in Table 1. The fasting group received slightly less saline infusion and had a lower saline infusion index; otherwise, there were no statistically significant differences in baseline parameters.

### 3.2. Left Ventricular Volumes and Ejection Fraction

**Figure 2:**
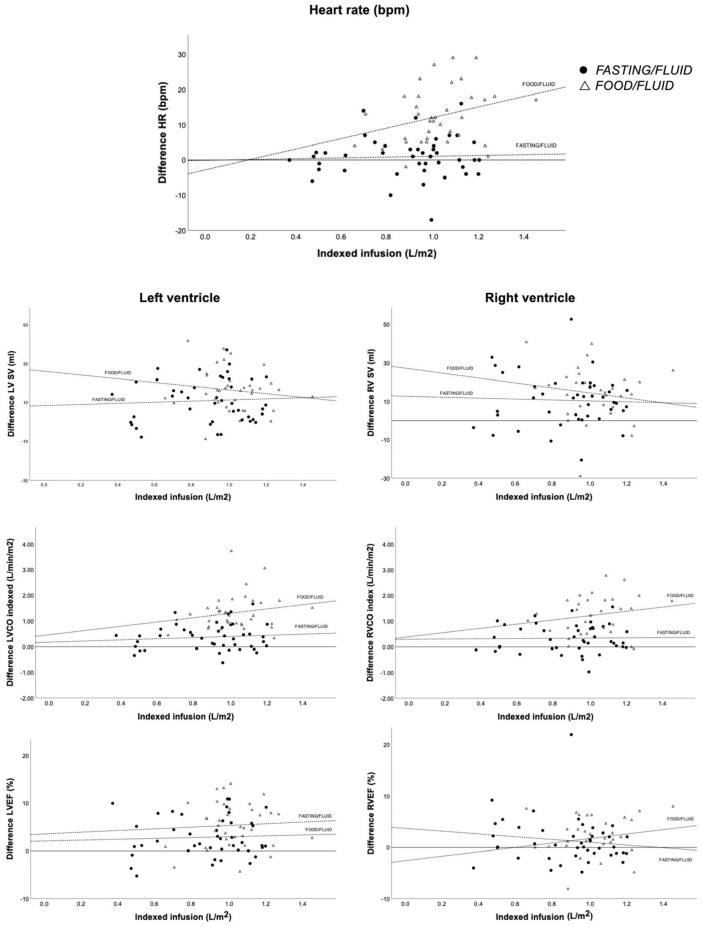
Relationship between various parameters and indexed infusion. Scatterplot with linear regression for both groups (fasting/fluid and food/fluid) demonstrates the relationship between indexed infusion and pre–post differences in heart rate, stroke volume, indexed cardiac output, and ejection fraction for both the left and right ventricles. CO: cardiac output; HR: heart rate; LV: left ventricle; LVEF: left ventricle ejection fraction; RV: right ventricle; RVEF: right ventricle ejection fraction; SV: stroke volume.

**Figure 3:**
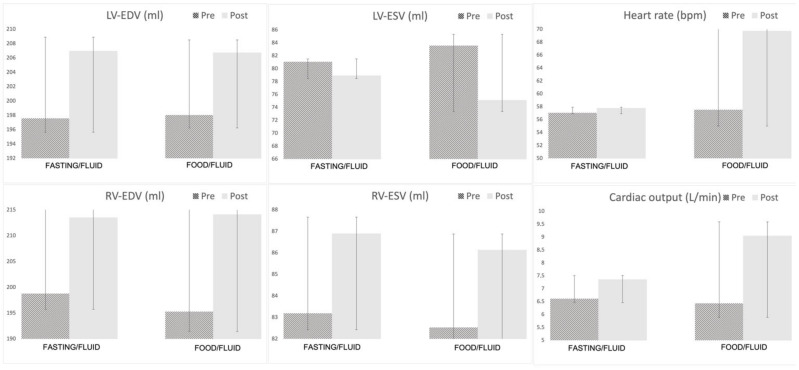
Mean values of various parameters. Bar chart demonstrating cardiovascular magnetic resonance mean values and standard deviation before and after fluid infusion and +/− food. EDV: end-diastolic volume; ESV: end-systolic volume; LV: left ventricle; RV: right ventricle.

LV volumes before and after saline infusion for both groups are presented in Table 2. During saline infusion, we observed an increase in LV-EDV, LV-SV, LV-CO, indexed LV-CO, and LV-EF in both groups (Figure 2). When comparing differences between the two groups during fluid infusion, the food group had significantly higher increases in LV-SV, LV-CO, indexed LV-CO, and LV-EF and a greater reduction in LV-ESV. In a multiple linear regression model including both indexed fluid infusion (L/m^2^) and oral intake of food (+/−), only food intake had a significant impact on LV-CO (Table 3). Indexed LV-CO showed a linear increase in the food group according to increased fluid infusion but only a very small increase in the fasting group (Figure 3).

### 3.3. Right Ventricular Volumes and Ejection Fraction

During fluid infusion, both RV-EDV and RV-SV increased significantly in both groups. The increase in RV-CO and indexed RV-CO were significantly higher in the food/fluid group compared to the fasting/fluid group (2.4 ± 1.4 vs. 0.7 ± 1.1; *p* < 0.001 and 1.2 ± 0.8 vs. 0.3 ± 0.5; *p* < 0.001). In the multiple linear regression model, only food intake had a significant impact on indexed RV-CO (Table 3).

### 3.4. Left Ventricular Strain and Feature Tracking

LV strain by TTE and FT by CMR are presented in Table 4. During fluid infusion, we observed significantly improved GLS and FT in both groups. When comparing the differences between the two groups, only FT by CMR was significantly improved.

### 3.5. Right Ventricular Strain and Feature Tracking

During fluid infusion, there were no significant changes in the fasting group. In the food group, we observed significantly increased RV-FT and RV strain in all segments. There was a trend towards an increase in RV strain of the free wall, but this increase was not significant.

### 3.6. Heart Rate

There was no difference in HR at baseline but significant differences after food intake. Post–pre differences were significantly higher in the food/fluid group (1 ± 6 vs. 12 ± 8, *p* < 0.001) (Table 2).

**Figure 4:**
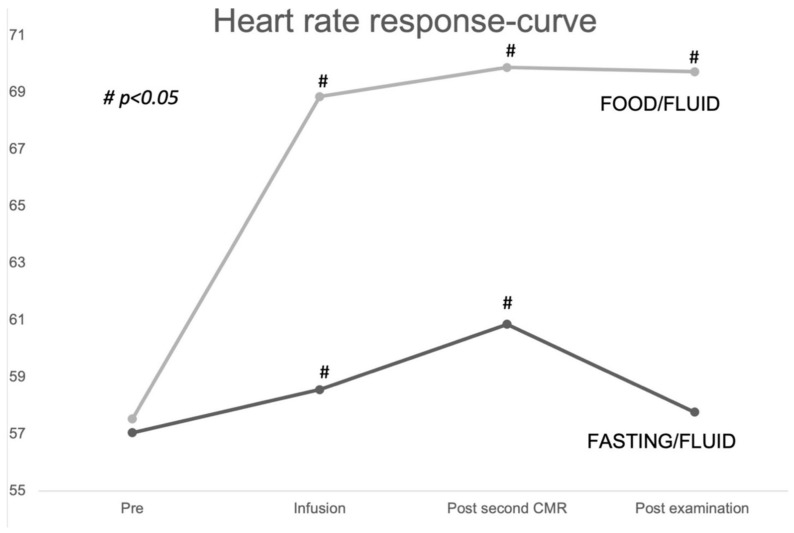
Heart rate response at four time points during the examination day. Line chart demonstrating the changes in heart rate during the examination day in the two subgroups. # indicating a significant difference from the pre-examination heart rate. Pre: pre-examination.

The effect of preload augmentation and food intake on HR at four different time points showed a significant increase after both fluid alone and fluid plus food. This effect decreased over time, ending with a non-significant post–pre difference in the fasting/fluid group. Figure 4 illustrates the changes in HR during the examination day.

### 3.7. Global Longitudinal Strain Deterioration According to Current Cardio-Oncology Guidelines

Depending on whether the ESC^21^ or ESMO22 guidelines are used, respectively, 17% (1 patient with an absolute GLS drop ≥ 5% and 13 patients with a relative GLS drop ≥ 12%) and 7% (6 patients with a GLS drop > 15%) of patients manifested clinically significant GLS deterioration when the post-examination was used as a baseline.

## 4. Discussion

In this study, we investigated the effects of food intake and preload augmentation on cardiac volumes and function assessed using feature tracking by CMR and GLS by echocardiography.

### 4.1. Our Most Important Findings Are

(1) CO and LV contractility (measured as LVEF, GLS, and CMR-FT) are significantly increased by preload augmentation and further increased by food intake.
(2) Fluid infusion and food intake may falsely improve GLS, which may affect follow-up.
(3) The LV and RV seem to act through different mechanisms when increasing CO: the LV through increased contractility and the RV through augmented EDV and ESV, which are indirectly driven by the effects of the LV.

### 4.2. Food Intake Recommendations before Cardiac Assessment

Cardiac assessment of hemodynamic and systolic function are important measures for diagnosis, prognosis, and decision-making in patients with, or at risk of, cardiac dysfunction. Currently, there are no widely accepted guidelines regarding fluid or food intake before cardiac assessments using echocardiography, CMR, or right heart catheterization [1,2,3,4].

Previous studies [6,26] found that LV-CO increased in healthy subjects after a meal and that the increase was driven by an increase in both SV and HR. The increase in LV-CO after a larger meal was greater and lasted longer. Another study [9] also found that LV-CO increased after food intake. However, this increase was only driven by increased HR. In this study, preload augmentation with or without food intake had a significant impact on SV, CO, and indexed CO in both LV and RV.

We found a significant increase in HR between the baseline and second CMR in both groups, with the fasting/fluid group reverting to baseline more rapidly and only the food/fluid group maintaining a persistently increased HR at the end of the study day. Similar patterns have also been demonstrated before [9,11,27].

### 4.3. Mechanisms to Increase Cardiac Output—Differences between LV and RV

We observed significantly increased CO with preload augmentation and significantly larger increases when adding food. However, the mechanism behind the increase seems different between the two ventricles. The LV responds to preload augmentation and food intake by increasing HR and contractility, with an attendant decrease in LVESV. The RV responds with increasing volumes, indicated by an increase in RVEDV and an unchanged/trend towards increasing RVESV (Figure 3). Our findings are consistent with previous studies showing that food intake increases HR, SV, and CO for the LV [6,7,8,9]. There is a paucity of data on the impact of food intake on the hemodynamics of the RV.

The physiological explanation for the different mechanisms to increase CO could be the thinner wall, lower volume-to-mass ratio, and smaller cardiomyocytes of the RV that make the RV more compliant and able to dilate [14]. In addition, the myocardial layers are different in the two ventricles. The LV has three distinct layers of aggregated myocytes; however, no proper middle layer is defined in the RV under normal physiological conditions. The subepicardial layer of the RV is arranged with more circumferential myofibers than the LV, and circumferential fibers only account for 20–30% of the ejection fraction, whereas helical fibers cause longitudinal strain and account for >60% of the ejection fraction [14,28,29]. The RV has reduced circumferential shortening compared to the LV [30]. The thinner wall, lower muscle mass, smaller size of the cardiomyocytes, reduced circumferential shortening, and/or the difference in myocardial layers and myofiber organization can possibly explain why RV reacts differently to preload augmentation and food intake than LV. In addition, the RV shows a distinctive, peristaltic-like contraction pattern [28], which might be less efficient at increasing contractility.

In our study, the RVEF increased when adding food intake to preload augmentation; however, the RVEF decreased with only preload. A possible explanation is that LV contraction contributes 20–40% of the RV SV and, thus, pulmonary flow. Therefore, increased LV contractility after food intake indirectly affects the RVEF [14].

### 4.4. The Clinical Impact of Variations in Global Longitudinal Strain in Follow-Up Patients

In all patients where serial measurements of cardiac function are necessary, it is important that all external conditions are equal at every examination to avoid inaccuracies. A theoretical example of this importance is in the field of cardio-oncology.

Cardiovascular diseases in patients with cancer are a major challenge since the considerable improvements in cancer treatment are at the cost of short- and long-term cardiovascular adverse effects. Cancer-therapy-related heart failure is a leading cause of morbidity and mortality in cancer patients [31,32]. There is consequently an increasing focus on cardiovascular monitoring and management of patients in treatment with cancer drugs. One current cardio-oncology guideline defines myocardial toxicity based on GLS as an absolute drop ≥5% or a relative drop ≥12%, whereas another defines it as a relative drop >15% [17,33,34]. For example, if we define the post-food examination as “baseline” and the pre-food examination as “post-chemotherapy” for the oncology patient, respectively, 17% or 7% of the patients in our study develop “cardiac dysfunction” according to the two current cardio-oncology guidelines [33,34]. Patients awaiting chemotherapy who eat before the first evaluation may receive a falsely better cardiac assessment. If the patient does not eat before the second evaluation after the initiation of chemotherapy, a normalization of the cardiac function may “mimic” cardiac dysfunction. This may potentially result in an inappropriate switch to less effective chemotherapy. Conversely, if the patient does not eat before the primary assessment but at the second assessment, this may impede the detection of clinical deterioration, resulting in continued cardiotoxic treatment without cardioprotective therapies and leading to a worsening in cardiac function. Both situations can predispose to suboptimal treatment [35].

The scenarios described above are relevant not only in oncocardiology but also in all other patients where multiple follow-up examinations are performed. The scenarios underline and support our recommendations that patients should not eat or drink before a cardiac evaluation, or at least ensure there is equivalent intake before all examinations.

### 4.5. Limitations

There are some limitations that need to be highlighted. *First*, the TTE and CMR were performed non-blind to food status. However, all examinations were anonymized and completely blinded during the post-processing analysis. *Second*, the meals in the food group consisted of both a standardized food intake and coffee/tea/soda with potential caffeine. The effects of food intake may wholly or partly represent the effects of caffeine or oral fluid intake. Our meal represents a usual lunch and was chosen to mimic a normal situation. For future studies, a standardized meal with exact energy intake and nutritional information, with/without caffeine, would be preferable. *Third*, CMR was always performed right before and right after fluids and food (when not fasting). Consequently, the second TTE was performed last, and there might be a risk that the effects of fluid or food have subsided. Table 2 demonstrates a significant improvement in LV-FT in the food group but no significant improvement in strain by TTE. Previous studies have shown that LV function and hemodynamics show a maximum increase 30–60 min after food, with most parameters returning to baseline within 2 h. Hence, in our study, there is a risk that the hemodynamic effects may have diminished at the second TTE [6,11,27].

## 5. Conclusions

Preload augmentation and food intake significantly impact the hemodynamic and cardiac functional parameters. This advocates for standardized recommendations regarding oral intake before cardiac assessment, for example, TTE, CMR, and right heart catheterization. We also demonstrate different approaches for the LV and RV to increase SV: for the LV by increased contractility, and for the RV by volume expansion.

## Figures and Tables

**Table 1:** Baseline characteristics of the study population (*n* = 82).

	Fasting/Fluid (*n* = 43)	Food/Fluid (*n* = 39)	*p*-Value
Age (years)	42 ± 15	45 ± 13	0.35
Male sex, n (%)	30 (70)	27 (70)	1.00
Height (m)	179 ± 9	178 ± 10	0.77
Weight (kg)	80 ± 16	82 ± 16	0.66
BMI (kg/m^2^)	25 ± 4	26 ± 4	0.47
BSA (m^2^)	2.0 ± 0.2	2.0 ± 0.2	0.82
Saline infusion (L)	1.7 ± 0.4	2.0 ± 0.3	<0.01
Saline infusion index (L/m^2^)	0.9 ± 0.2	1.0 ± 0.2	<0.01
Cardiac disease, n (%)	23 (54)	17 (44)	0.29
Risk factors			
Diabetes, n (%)	1 (2)	2 (5)	0.51
Hypertension, n (%)	8 (19)	9 (23)	0.62
Stroke or TIA, n (%)	1 (3)	0	0.30
Previous/current smoker, n (%)	21 (49)	14 (36)	0.24
Left ventricular geometry			
Normal, n (%)	15 (35)	16 (41)	0.57
Dilated, n (%)	13 (30)	10 (26)	0.65
Hypertrophied, n (%)	8 (19)	5 (13)	0.48
Dilated + Hypertrophied, n (%)	7 (16)	8 (21)	0.63

Comparison of different baseline characteristics in the two subgroups: fasting/fluid and food/fluid. BMI: body mass index; BSA: body surface area; TIA: transient ischemic attack.

**Table 2:** Cardiac volumes by cardiac magnetic resonance: pre/post saline infusion and divided into fasting/fluid and food/fluid groups.

	Fasting/Fluid *n* = 43			Food/Fluid *n* = 39			Pre vs. Pre	Diff (Post–Pre)		Diff vs. Diff
	Pre	Post	*p*	Pre	Post	*p*	*p*	Fasting/Fluid	Food/Fluid	*p*
SBP (mmHg)	124 ± 18	127 ± 19	<0.05	129 ± 16	130 ± 17	NS	NS	3 ± 9	1 ± 10	NS
DBP (mmHg)	73 ± 12	76 ± 12	<0.01	78 ± 13	79 ± 11	NS	<0.05	3 ± 6	2 ± 9	NS
HR (bpm)	57 ± 10	57 ± 11	NS	57 ± 10	70 ± 11	<0.001	NS	1 ± 6	12 ± 8	<0.001
LV-EDV (mL)	198 ± 59	207 ± 57	<0.001	198 ± 64	207 ± 58	<0.001	NS	9 ± 13	9 ± 12	NS
RV-EDV (mL)	200 ± 47	214 ± 50	<0.001	195 ± 43	212 ± 45	<0.001	NS	14 ± 16	18 ± 14	NS
LV-ESV (mL)	81 ± 29	79 ± 28	NS	82 ± 35	75 ± 32	<0.001	NS	−2 ± 10	−7 ± 11	<0.05
RV-ESV (mL)	84 ± 26	87 ± 31	NS	82 ± 23	86 ± 25	NS	NS	3 ± 13	4 ± 11	NS
LV-SV (mL)	117 ± 34	128 ± 33	<0.001	115 ± 33	132 ± 32	<0.001	NS	11 ± 11	16 ± 11	<0.05
RV-SV (mL)	116 ± 25	127 ± 26	<0.001	113 ± 24	127 ± 26	<0.001	NS	11 ± 13	14 ± 14	NS
LV-CO (L/min)	6.6 ± 2.1	7.4 ± 2.1	<0.001	6.5 ± 1.4	9.0 ± 2.3	<0.001	NS	0.7 ± 1.0	2.6 ± 1.3	<0.001
RV-CO (L/min)	6.6 ± 1.9	7.3 ± 1.8	<0.001	6.3 ± 1.4	8.8 ± 1.7	<0.001	NS	0.7 ± 1.1	2.4 ± 1.4	<0.001
LV-CO index (L/min/m^2^)	3.3 ± 0.9	3.7 ± 1.0	<0.001	3.3 ± 0.6	4.6 ± 1.1	<0.001	NS	0.4 ± 0.5	1.3 ± 0.7	<0.001
RV-CO-index (L/min/m^2^)	3.3 ± 0.8	3.7 ± 0.8	<0.001	3.2 ± 0.6	4.5 ± 0.8	<0.001	NS	0.3 ± 0.5	1.2 ± 0.8	<0.001
LVEF(%)	59 ± 6	62 ± 5	<0.001	59 ± 6	64 ± 6	<0.001	NS	3 ± 4	5 ± 5	<0.05
RVEF(%)	59 ± 6	60 ± 6	NS	58 ± 5	60 ± 5	<0.05	NS	1 ± 5	2 ± 5	NS

Comparison of different pre and post-parameters in the two subgroups fasting/fluid and food/fluid. Diff: differences; SBP: systolic blood pressure; DBP: diastolic blood pressure; HR: heart rate; LV-EDV: left ventricular end-diastolic volume; LV-ESV: left ventricular end-systolic volume; LV-SV: left ventricular stroke volume; LV-CO: left ventricular cardiac output; LVEF: left ventricular ejection fraction; RV-EDV: right ventricular end-diastolic volume; RV-ESV: right ventricular end-systolic volume; RV-SV: right ventricular stroke volume; RV-CO: right ventricular cardiac output; RVEF: right ventricular ejection fraction.

**Table 3:** Multiple linear regression analysis.

	Model	Food Intake		Indexed Fluid Infusion (L/m^2^)	
	*p*	B	*p*	B	*p*
LV-CO (L/min)	<0.001	1.8	<0.001	0.5	NS
LV-CO index (L/min/m^2^)	<0.001	0.9	<0.001	0.4	NS
RV-CO-index (L/min/m^2^)	<0.001	1.7	<0.001	0.4	NS
RV-CO-index (L/min/m^2^)	<0.001	0.9	<0.001	0.3	NS
LV-FT (%)	<0.001	−1.1	<0.01	−1.9	NS
GLS (%)	<0.05	−0.3	NS	−2.3	<0.05
GLS_basal_ (%)	<0.05	−0.3	NS	−2.0	<0.05
GLS_mid_ (%)	<0.05	−0.1	NS	−1.8	<0.05
GLS_apical_ (%)	NS	−0.6	NS	−3.4	NS
RV-FT (%)	NS	0.9	NS	1.3	NS
RV-strain_all_ (%)	<0.05	−2.9	<0.01	1.2	NS
RV-strain_freewall_ (%)	NS	−1.1	NS	−2.1	NS

Models combining the effects of food intake and fluid infusion on different parameters of the left and right ventricles. B: beta; LV-CO: left ventricular cardiac output; LV-CO index: left ventricular cardiac output indexed; LV-FT: left ventricular feature tracking; GLS: global longitudinal; RV-CO: right ventricular cardiac output; RV-CO-index: right ventricular cardiac output indexed; RV-FT: right ventricular feature tracking; RV-strain: right ventricular strain.

**Table 4:** Comparison of pre and post-values of feature tracking and global longitudinal strain in the two subgroups.

	Fasting/Fluid *n* = 43			Food/Fluid *n* = 39			Pre vs. Pre	Diff (Post–Pre)		Diff vs. Diff
	Pre	Post	*p*	Pre	Post	*p*	*p*	Fasting/Fluid	Food/Fluid	*p*
LV-FT (%)	−19.0 ± 2.9	−19.6 ± 3.5	<0.01	−19.1 ± 2.5	−21.0 ± 3.5	<0.001	NS	−0.6 ± 1.3	−2.0 ± 2.3	<0.01
GLS (%)	−18.8 ± 3.6	−19.7 ± 4.0	<0.001	−18.9 ± 3.0	−20.4 ± 3.4	<0.001	NS	−0.9 ± 1.5	−1.5 ± 1.8	NS
GLS_basal_ (%)	−17.9 ± 2.8	−18.3 ± 3.0	0.10	−17.4 ± 3.1	−18.3 ± 3.6	<0.001	NS	−0.4 ± 1.7	−1.0 ± 1.7	NS
GLS_mid_ (%)	−18.5 ± 3.6	−19.5 ± 4.0	<0.001	−18.8 ± 3.0	−20.0 ± 3.5	<0.001	NS	−1.0 ± 1.5	−1.3 ± 1.4	NS
GLS_apical_ (%)	−20.1 ± 5.6	−21.3 ± 6.0	<0.01	−20.8 ± 4.1	−23.0 ± 4.5	<0.001	NS	−1.2 ± 2.6	−2.2 ± 3.8	NS
RV-FT (%)	−26.2 ± 3.9	−26.9 ± 3.4	NS	−26.0 ± 3.3	−27.7 ± 3.1	<0.01	NS	−0.6 ± 3.0	−1.7 ± 3.1	NS
RV-strain_all_ (%)	−21.9 ± 2.8	−21.9 ± 2.9	NS	−20.8 ± 2.5	−23.5 ± 2.8	<0.01	<0.05	0.1 ± 2.0	−2.7 ± 1.6	<0.01
RV-strain_freewall_ (%)	−27.4 ± 3.5	−27.2 ± 3.8	NS	−25.3 ± 3.7	−26.3 ± 4.6	NS	NS	0.3 ± 2.9	−1.0 ± 3.5	NS

Feature tracking was assessed using cardiovascular magnetic resonance, and the global longitudinal strain was assessed using echocardiography: Pre/post-saline infusion, divided by fasting and food intake. Diff: differences; LV-FT: left ventricular feature tracking; GLS: global longitudinal strain; RV-FT: right ventricular feature tracking; RV: right ventricular.

## Data Availability

The data that support the findings of this study are available on request to the corresponding author.
